# Circular RNA HIPK3 downregulation mediates hydrogen peroxide-induced cytotoxicity in human osteoblasts

**DOI:** 10.18632/aging.102674

**Published:** 2020-01-18

**Authors:** Jinqian Liang, Ying-chao Shen, Xiang-yang Zhang, Chong Chen, Hong Zhao, Jianhua Hu

**Affiliations:** 1 Department of Orthopedic Surgery, Peking Union Medical College Hospital, Beijing, China; 2 Department of Orthopaedics, Changshu Hospital of Traditional Chinese Medicine, Changshu, China; 3 Department of Orthopaedics, Tongren Hospital, Shanghai Jiao Tong University School of Medicine, Shanghai, China

**Keywords:** circular RNA HIPK3, hydrogen peroxide, osteoblasts, microRNA-124, oxidative injury

## Abstract

Hydrogen peroxide (H_2_O_2_) induces oxidative injury to human osteoblasts. The expression and potential function of circular RNA HIPK3 (circHIPK3) in H_2_O_2_-treated human osteoblasts were tested. We show that H_2_O_2_ significantly downregulated circHIPK3 in OB-6 cells and primary human osteoblasts. Furthermore, circHIPK3 levels were decreased in the necrotic femoral head tissues of dexamethasone-treated patients. In OB-6 osteoblastic cells and primary human osteoblasts, forced overexpression of circHIPK3 by a lentiviral construct alleviated H_2_O_2_-induced viability reduction, cell death and apoptosis. Contrarily, circHIPK3 silencing by targeted shRNA potentiated H_2_O_2_-induced cytotoxicity in OB-6 cells and primary human osteoblasts. Moreover, circHIPK3 downregulation by H_2_O_2_ induced miR-124 accumulation in OB-6 cells and primary human osteoblasts. On the contrary, miR-124 inhibition by transfection of the miR-124 inhibitor protected human osteoblasts from H_2_O_2_. Importantly, forced overexpression of miR-124 by transfection of the miR-124 mimic induced significant cytotoxicity in OB-6 cells and primary human osteoblasts. H_2_O_2_ downregulated miR-124’s targets, cyclin dependent kinase 6 and Rho-Associated Protein Kinase 1, in human osteoblasts. In conclusion circHIPK3 downregulation mediates H_2_O_2_-induced cytotoxicity in human osteoblasts.

## INTRODUCTION

In the pathogenesis of osteoporosis and osteonecrosis, increased reactive oxygen species (ROS) production and oxidative injury will lead to severe damage to human osteoblasts and bone cells [1–4]. To the cultured human osteoblasts or osteoblastic cells hydrogen peroxide (H_2_O_2_) was added, as an *in vitro* cellular model of osteoporosis/osteonecrosis [5–8]. H_2_O_2_ induces profound oxidative stress, protein damage, lipid peroxidation and DNA breaks in human osteoblasts, leading to cell death and apoptosis. Further understanding the pathological mechanisms of H_2_O_2_-induced osteoblast injury is important for the development of possible intervention strategies [5–8].

Circular RNAs (circRNAs) are a large family of conserved and stable non-coding RNAs (ncRNAs) exclusively in the cytoplasm of eukaryotic cells [9, 10]. Compared with linear RNAs, circRNAs have covalently-closed loop structures, but without a free 3′ or 5′ end nor poly-adenylated tails [9, 10]. circRNAs function as microRNA (miRNA) sponges to sequester and competitively inhibit miRNA expression and activity [9, 10]. The potential functions of circRNAs in the pathogenesis of osteoporosis and osteonecrosis have not been extensively studied.

Derived from homeodomain-interacting protein kinase 3 (*HIPK3*) gene Exon2, the circular RNA HIPK3 (circHIPK3) has the sequence length of 1099 base-pair [11]. circHIPK3 could possibly exert pro-survival functions in a number of cancer cells, partially mediated through as sponges of cancer-suppressive miRNAs [11–13]. A very recent study has shown that circHIPK3 levels are downregulated in high glucose (HG)-treated human umbilical vein endothelial cells (HUVECs) and in primary human aortic endothelial cells (HAECs) from the diabetic patients [14]. More importantly, circHIPK3 downregulation mediated *in vitro* endothelial cell injury by HG [14]. The results of the current study will show that H_2_O_2_ downregulates circHIPK3 to promote human osteoblast cell death and apoptosis.

## RESULTS

### H_2_O_2_ downregulates circHIPK3 in human osteoblasts

**Figure 1:**
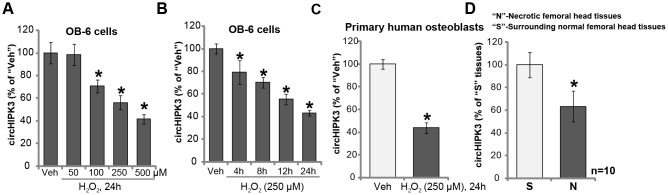
**H_2_O_2_ downregulates circHIPK3 in human osteoblasts.** OB-6 human osteoblastic cells or the primary human osteoblasts were treated with hydrogen peroxide (H_2_O_2_, at applied concentrations) and cultured for indicated time periods, relative circHIPK3 expression was tested by qPCR (**A**–**C**) qPCR analysis of the relative circHIPK3 expression in the surgery-isolated femoral head tissues (both normal and necrotic) from ten (10) different dexamethasone-treated patients (**D**) “Veh” stands for vehicle control (PBS, same for all Figures). Quantified values were mean ± standard deviation (SD). * *P* < 0.05 vs. “Veh” treatment (A–C) * *P* < 0.05 vs. “S” tissues (surrounding normal femoral head tissues) (**D**; n=10). Experiments were repeated three times, with similar results obtained.

We first tested the potential effect of H_2_O_2_ on the expression of circHIPK3 in human osteoblasts. The differentiated, osteoblast-like human OB-6 cells [15–17] were treated with H_2_O_2_. qPCR testing circHIPK3 expression confirmed that H_2_O_2_ dose-dependently downregulated circHIPK3 in OB-6 osteoblastic cells (Figure 1A). The levels of circHIPK3 decreased to 98.55 ± 9.39%, 70.68 ± 5.58%, 56.30 ± 6.23% and 41.59 ± 4.10% of control level, following 50 μM, 100 μM, 250 μM and 500 μM of H_2_O_2_ treatment, respectively (Figure 1A). Furthermore, H_2_O_2_-induced circHIPK3 downregulation was time-dependent (Figure 1B). In OB-6 cells circHIPK3 downregulation started as early as 4 hours (4h) following H_2_O_2_ treatment (250 μM), and it lasted for at least 24h (Figure 1B). In the primary human osteoblasts, significant circHIPK3 downregulation was detected as well following H_2_O_2_ treatment (250 μM, 24h) (Figure 1C). Significantly, circHIPK3 expression levels were decreased in the necrotic femoral head tissues of dexamethasone-treated patients (Figure 1D). While its levels in surrounding normal femoral head tissues are relatively high (Figure 1D).

### Forced overexpression of circHIPK3 alleviates H_2_O_2_-induced death and apoptosis in human osteoblasts

**Figure 2:**
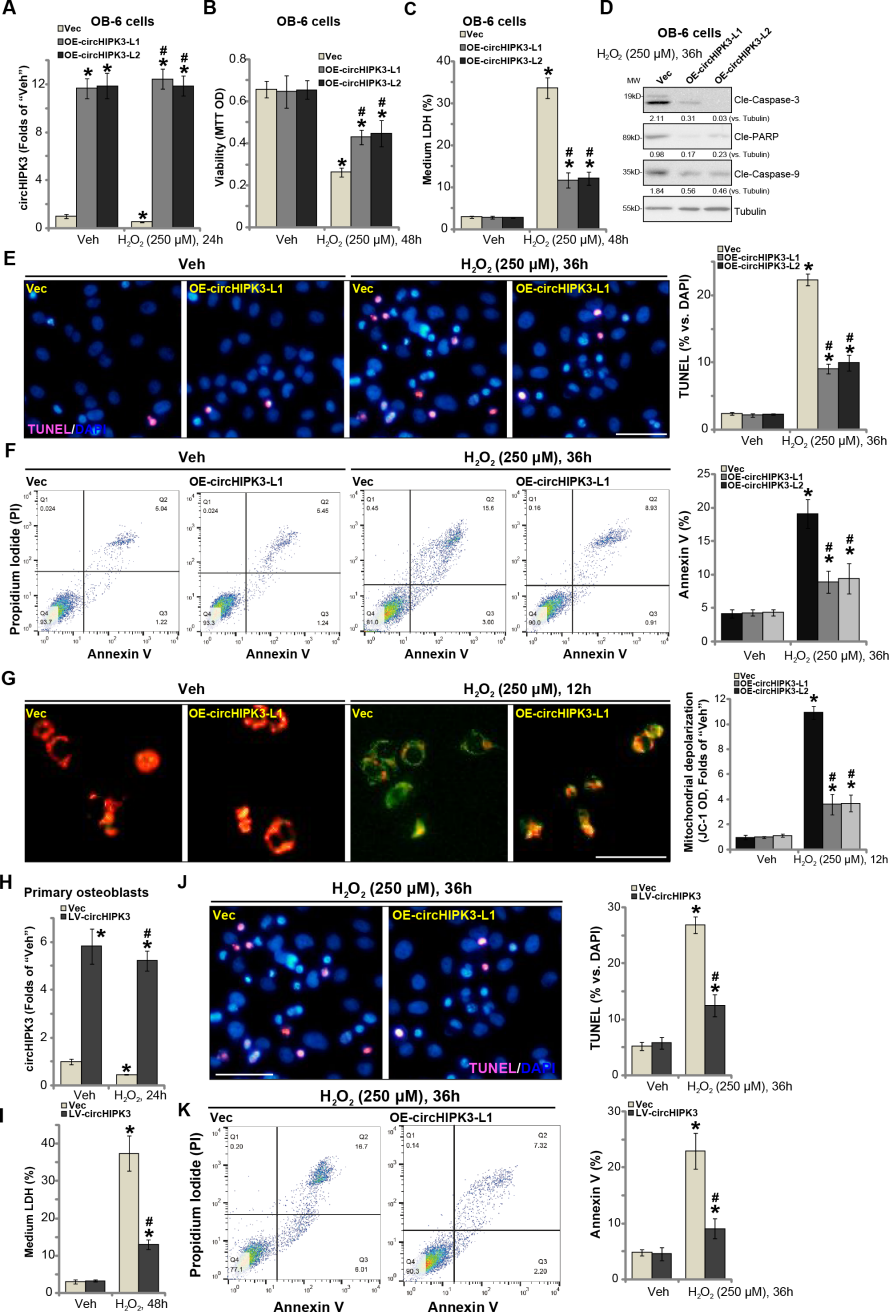
**Forced overexpression of circHIPK3 alleviates H_2_O_2_-induced death and apoptosis in human osteoblasts.** OB-6 human osteoblastic cells were infected with circHIPK3-expressing lentivirus (“LV-circHIPK3”) or control lentivirus (with empty vector, “Vec”), following puromycin selection stable cell lines were established (“OE-circHIPK3-L1/2”). Cells were treated with hydrogen peroxide (H_2_O_2_, 250 μM) and cultured for the applied time periods, relative circHIPK3 expression was tested by qPCR assay (**A**); Cell viability (**B**), cell death (**C**), cell apoptosis (**D**–**F**) and mitochondrial depolarization (**G**) were tested by the assays mentioned in the text, and results were quantified. The primary human osteoblasts were infected with “LV-circHIPK3” or “Vec” for 24h, then treated with hydrogen peroxide (H_2_O_2_, 250 μM) and cultured for the applied time periods, relative circHIPK3 expression and cell death were tested by qPCR (**H**) and LDH release (**I**) assays, respectively; Cell apoptosis was tested by TUNEL staining (**J**) and Annexin V-FACS (**K**) assays. Expression of the listed proteins was quantified and normalized to the loading control protein (β-) Tubulin (**D**). “MW” stands for molecular weight (Same for all Figures). Quantified values were mean ± standard deviation (SD, n=5). * *P* < 0.05 vs. “Veh” treatment of “Vec” cells. ^#^ *P* < 0.05 vs. H_2_O_2_ treatment of “Vec” cells. Experiments were repeated five times, with similar results obtained. Bar=100 μm (**E**, **G** and **J**).

The results in Figure 1 indicate a potential activity of circHIPK3 in H_2_O_2_-induced cytotoxicity. To test this hypothesis, circHIPK3-expressing lentivirus (“LV-circHIPK3”, from Dr. Lu at Nanjing University of Traditional Chinese Medicine [14]) was transduced to OB-6 osteoblastic cells. Following selection by puromycin two stable cell lines with LV-circHIPK3 were established: “OE-circHIPK3-L1 and OE-circHIPK3-L2”. Analyzing circHIPK3 expression, by qPCR, confirmed that circHIPK3 levels increased over ten folds in the LV-circHIPK3-expressing OB-6 cells (Figure 2A), even with H_2_O_2_ treatment (Figure 2A).

It has been previously shown that H_2_O_2_ could induce both programmed necrosis and apoptosis in human osteoblasts and osteoblastic cells [18, 19]. Significantly, H_2_O_2_-induced cell viability (MTT OD) reduction (Figure 2B) and death (increased medium LDH release, Figure 2C) were significantly inhibited in circHIPK3-overexpressed stable OB-6 cells. Furthermore, H_2_O_2_-induced apoptosis activation in OB-6 cells was attenuated by circHIPK3 overexpression as well (Figure 2D and 2E). Apoptosis activation in H_2_O_2_-treated OB-6 cells was evidenced by cleavages of caspase-3, caspase-9 and ploy ADP ribose polymerase (PARP) (Figure 2D) as well as the increased nuclear TUNEL staining ratio (Figure 2E). Furthermore, ectopic overexpression of circHIPK3 largely inhibited H_2_O_2_-induced increase in Annexin V staining (Figure 2F), further supporting the anti-apoptosis activity by circHIPK3. Additionally, H_2_O_2_ treatment in vector control OB-6 cells induced mitochondrial depolarization, tested by JC-1 green intensity increase (Figure 2G). The actions by H_2_O_2_ were again inhibited in circHIPK3-overexpressed OB-6 cells (Figure 2G).

In the primary human osteoblasts, LV-circHIPK3 similarly resulted in an increase of circHIPK3 expression, regardless of H_2_O_2_ stimulation (Figure 2H). H_2_O_2_-induced cell death (Figure 2I, tested by LDH medium release) was significantly alleviated by LV-circHIPK3 in the primary osteoblasts. Furthermore, circHIPK3 overexpression potently inhibited H_2_O_2_-induced apoptosis activation, decreasing cell numbers with positive TUNEL (Figure 2J) and Annexin V (Figure 2K) staining. Together, these results showed that forced overexpression of circHIPK3 alleviated H_2_O_2_-induced death and apoptosis in human osteoblasts.

### circHIPK3 silencing potentiates H_2_O_2_-induced death and apoptosis in human osteoblasts

**Figure 3:**
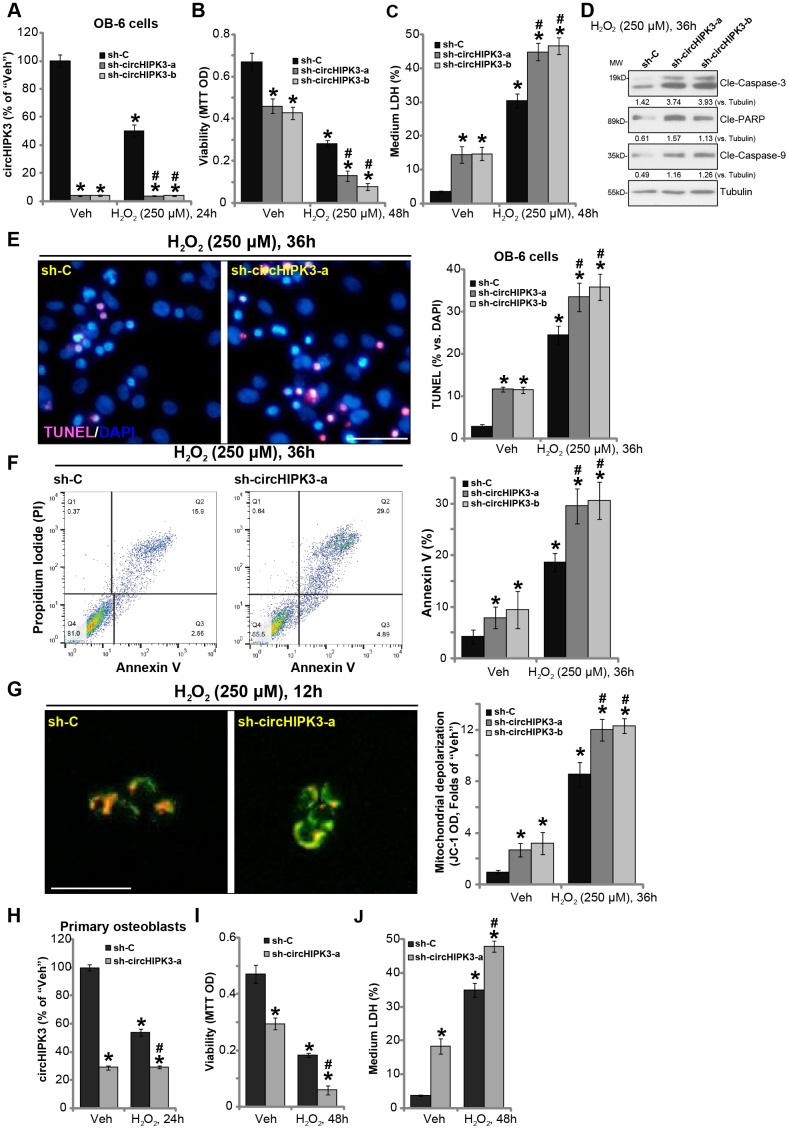
**circHIPK3 silencing potentiates H_2_O_2_-induced death and apoptosis in human osteoblasts.** OB-6 human osteoblastic cells were transfected with the lentiviral circHIPK3 shRNA (“sh-circHIPK3-a/b”, with non-overlapping sequences) or control shRNA lentivirus (“sh-C”), following puromycin selection the stable cells were established. Cells were treated with hydrogen peroxide (H_2_O_2_, 250 μM) and cultured for the applied time periods, relative circHIPK3 expression was tested by qPCR assay (**A**); Cell viability (**B**), cell death (**C**), cell apoptosis (**D**–**F**) and mitochondrial depolarization (**G**) were tested by the assays mentioned in the text, and results were quantified. The primary human osteoblasts were infected with “sh-circHIPK3-a” lentivirus or “sh-C” lentivirus for 24h, and then treated with hydrogen peroxide (H_2_O_2_, 250 μM) and cultured for the applied time periods, relative circHIPK3 expression, cell viability and death were tested by qPCR (**H**), MTT (**I**), and LDH release assay (**J**), respectively. Expression of the listed proteins was quantified and normalized to the loading control protein (β-) Tubulin (**D**). Quantified values were mean ± standard deviation (SD, n=5). * *P* < 0.05 vs. “Veh” treatment of “sh-C” cells. ^#^ *P* < 0.05 vs. H_2_O_2_ treatment of “sh-C” cells. Experiments were repeated five times, with similar results obtained. Bar=100 μm (**E** and **G**).

Previous studies have indicated that circHIPK3 is important for cell survival [14, 20]. We therefore proposed that circHIPK3 silencing could possibly intensify H_2_O_2_-induced cytotoxicity in human osteoblasts. Two lentiviral shRNAs, against non-overlapping sequences of circHIPK3 (“sh-circHIPK3-a/b”), were individually transduced to OB-6 cells. Following selection of puromycin stable cell lines were established. qPCR results, in Figure 3A, confirmed that the applied circHIPK3 shRNAs resulted in over 90% reduction of circHIPK3 expression in OB-6 cells, regardless of H_2_O_2_ treatment. Significantly, circHIPK3 silencing, by sh-circHIPK3-a/b, induced OB-6 cell viability (MTT OD) reduction (Figure 3B) and cell death (increased medium LDH release, Figure 3C). Importantly, H_2_O_2_-induced cytotoxicity was potentiated in circHIPK3-silenced OB-6 cells (Figure 3B and 3C). Moreover, in stable OB-6 cells bearing the circHIPK3 shRNAs, H_2_O_2_-induced apoptosis activation was significantly exacerbated as well (Figure 3D-3F). Cell apoptosis was tested by caspase-3, caspase-9 and PARP cleavages (Figure 3D) as well as increased nuclear TUNEL staining (Figure 3E) and Annexin V staining (Figure 3F). H_2_O_2_-induced mitochondrial depolarization, shown by JC-1 green intensity increase, was also intensified with circHIPK3 silencing (Figure 3G).

In the primary human osteoblasts, the lentiviral circHIPK3 shRNA (“sh-circHIPK3-a”) similarly induced circHIPK3 downregulation (Figure 3H), cell viability reduction (Figure 3I) and death (Figure 3J). Moreover, circHIPK3 shRNA potentiated H_2_O_2_-induced cytotoxicity in human osteoblasts (Figure 3I and 3J). These results show that circHIPK3 silencing potentiated H_2_O_2_-induced cytotoxicity in human osteoblasts.

### miR-124 inhibition attenuates H_2_O_2_-induced cytotoxicity in human osteoblasts

**Figure 4:**
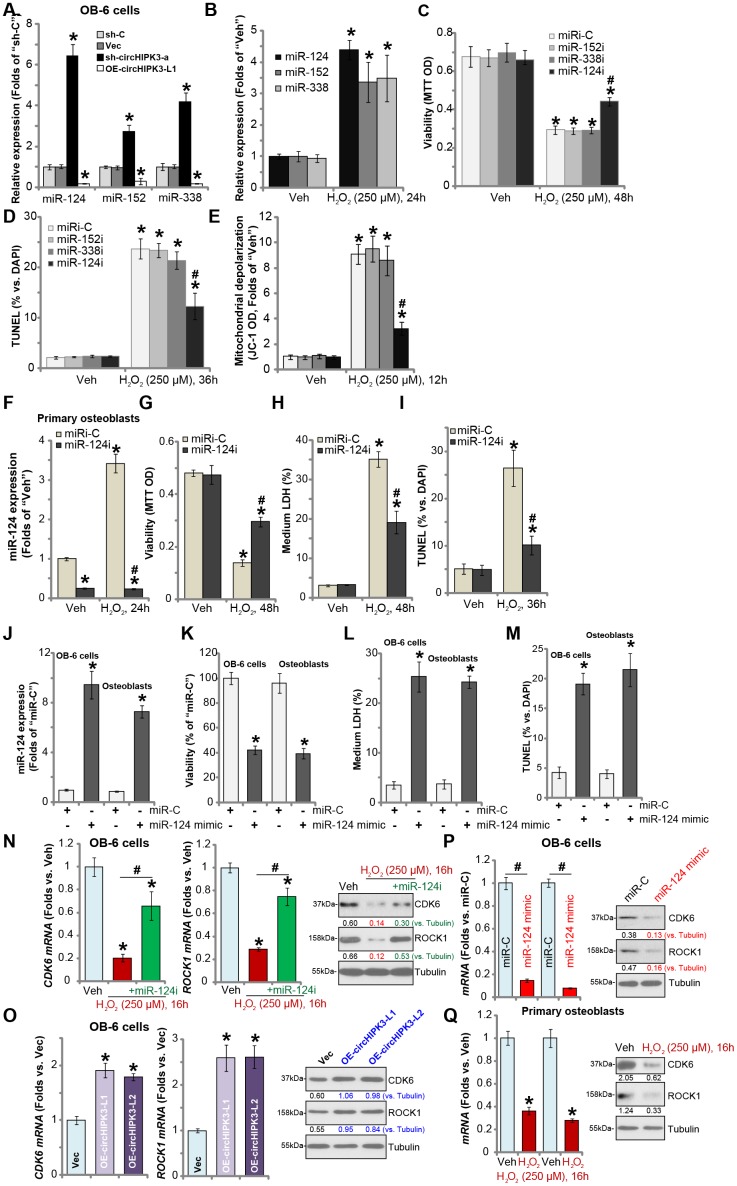
**miR-124 inhibition attenuates H_2_O_2_-induced cytotoxicity in human osteoblasts.** OB-6 human osteoblastic cells were transfected with circHIPK3-expressing lentivirus (“LV-circHIPK3”) or control lentivirus (with empty vector, “Vec”), as well as lentiviral circHIPK3 shRNA (“sh-circHIPK3-a”) or control shRNA lentivirus (“sh-C”), expression of listed microRNAs (miR-124, miR-152 and miR-338) was tested by qPCR (**A**). OB-6 cells were treated with hydrogen peroxide (H_2_O_2_, 250 μM) and cultured for 24h, expression of listed microRNAs (miR-124, miR-152 and miR-338) was tested by qPCR (**B**). OB-6 cells were transfected with miR-124 inhibitor (“miR-124i”, 500 nM), miR-152 inhibitor (“miR-152i”, 500 nM), miR-338 (“miR-338i”, 500 nM) or the non-sense control miRNA inhibitor (“miRi-C”) for 24h, followed by hydrogen peroxide (H_2_O_2_, 250 μM) treatment, cell viability and apoptosis were tested by MTT (**C**) and TUNEL staining (**D**), respectively. Mitochondrial depolarization was tested by JC-1 assay (**E**). The primary human osteoblasts were transfected with 500 nM of miR-124i or the miRi-C for 24h, followed by hydrogen peroxide (H_2_O_2_, 250 μM) treatment for indicated time periods, relative miR-124 expression (**F**), cell viability (**G**), cell death (**H**) and apoptosis (**I**) were tested by qPCR, MTT, LDH release and TUNEL staining assays, respectively. OB-6 cells or the primary human osteoblasts (“Osteoblasts”) were transfected with 500 nM of the miR-124 mimic or miR non-sense control (“miR-C”) for 48h, relative miR-124 expression (**J**), cell viability (**K**), cell death (**L**) and apoptosis (**M**) were tested similarly. OB-6 cells were transfected with the miR-124 inhibitor (“miR-124i”, 500 nM) for 24h, followed by H_2_O_2_ (250 μM) treatment for 16h, expression of listed genes was shown (**N**); The mRNA and protein expression of CDK6 and ROCK1 in stable OB-6 osteoblastic cells, with circHIPK3-expressing lentivirus (“OE-circHIPK3-L1/2”) or control lentivirus (with empty vector, “Vec”), was shown (**O**); OB-6 cells were transfected with 500 nM of the miR-124 mimic or miR non-sense control (“miR-C”) for 48h, with expression of listed genes examined (**P**). The primary human osteoblasts with or without H_2_O_2_ (250 μM, 16h) treatment were examined for the listed genes (**Q**). Quantified values were mean ± standard deviation (SD, n=5). * *P* < 0.05 vs. “sh-C” cells (**A**); * *P* < 0.05 vs. “Veh” treatment (**B**–**I**, **N** and **Q**). * *P* < 0.05 vs. “Vec” cells (**O**). ^#^ *P* < 0.05 vs. H_2_O_2_ treatment of “miRi-C” cells (**C**–**H**); * *P* < 0.05 vs. “miR-C” cells (J-M). ^#^ *P* < 0.05 (**N** and **P**). Experiments were repeated three times, with similar results obtained.

circRNAs sponge target miRNAs. It has been previously shown that circHIPK3 physically associates and degrads multiple microRNAs, including miR-124, miR-152 and miR-338 [11, 13]. As shown in Figure 4A, expression levels of miR-124, miR-152 and miR-338 were significantly increased in stable OB-6 cells bearing circHIPK3 shRNA (“sh-circHIPK3-a”, see Figure 3), but decreased in circHIPK3-overexpressed OB-6 cells (“OE-circHIPK3-L1”, see Figure 2). Moreover, H_2_O_2_ treatment, which downregulated circHIPK3, induced accumulations of miR-124, miR-152 and miR-338 in OB-6 cells (Figure 4B). In OB-6 cells transfection of the miR-124 inhibitor (“miR-124i”) potently inhibited H_2_O_2_-induced viability reduction (Figure 4C) and apoptosis activation (Figure 4D). On the contrary, miR-152 inhibitor (“miR-152i”) and miR-338 inhibitor (“miR-338i”) had no significant effect on H_2_O_2_-induced cytotoxicity (Figure 4C and 4D). H_2_O_2_-induced mitochondrial depolarization, or JC-1 green intensity increase, was largely attenuated by miR-124i (Figure 4E), while other miR inhibitors were ineffective (Figure 4E). In the primary human osteoblasts H_2_O_2_ similarly induced miR-124 accumulation, reversed by miR-124i (Figure 4F). Furthermore, H_2_O_2_-induced viability reduction (Figure 4G), cell death (Figure 4H) and apoptosis (Figure 4I) were significantly attenuated by miR-124i.

Based on the results we proposed that H_2_O_2_-induced downregulation of circHIPK3 caused miR-124 accumulation, mediating osteoblast cell death and apoptosis. Thus, forced expression of miR-124 should induce the similar action of H_2_O_2_. To test this hypothesis, the miR-124 mimic was transfected to OB-6 cells and primary human osteoblasts, resulting in significant increase in miR-124 expression (Figure 4J). Significantly, the miR-124 mimic induced viability reduction (Figure 4K), cell death (Figure 4L) and apoptosis (Figure 4M) in OB-6 cells and primary human osteoblasts.

We also tested the potential effect of H_2_O_2_ on the expression of miR-124’s targets, including cyclin dependent kinase 6 (CDK6) and Rho-Associated Protein Kinase 1 (ROCK1) [20–23]. qPCR and Western blotting assays were performed in OB-6 osteoblastic cells. Results showed that mRNA and protein expression of CDK6 and ROCK1 was significantly decreased following H_2_O_2_ treatment (Figure 4N), which was attenuated by miR-124i (Figure 4N). On the contrary, LV-circHIPK3-induced ectopic overexpression of circHIPK3, which depleted miR-124 (see Figure 2), resulted in upregulation of CDK6 and ROCK1 expression (both mRNA and protein, Figure 4O). Importantly, forced expression of miR-124, by transfection of miR-124 mimic, downregulated CDK6 and ROCK1 in OB-6 cells (Figure 4P). In the primary human osteoblasts H_2_O_2_ treatment resulted in downregulation of the two miR-124 targets (Figure 4Q). These results demonstrated that H_2_O_2_ downregulated miR-124’s targets, CDK6 and ROCK1, in human osteoblasts, further supporting the function of miR-124 in H_2_O_2_-induced cytotoxicity in osteoblasts.

## DISCUSSION

CircRNAs are formed from exon transcripts through non-linear reverse splicing or gene re-arrangements [9, 10, 24]. Dysregulation of circRNAs could be important for oxidative stress-induced osteoblast injury and pathogenesis of osteoporosis/osteonecrosis. The results of the present study show that circHIPK3 is downregulated in the necrotic femoral head tissues of dexamethasone-treated human patients, indicating a possible association between circHIPK3 reduction and pathophysiology of femoral head necrosis.

*In vitro* results of this study show that H_2_O_2_ downregulated circHIPK3 in OB-6 cells and primary human osteoblasts. Importantly, forced overexpression of circHIPK3, by a lentiviral construct, alleviated H_2_O_2_-induced viability reduction, cell death and apoptosis. Contrarily, circHIPK3 silencing by targeted shRNAs potentiated H_2_O_2_-induced cytotoxicity in OB-6 cells and primary human osteoblasts. These results imply that circHIPK3 downregulation mediates H_2_O_2_-induced cytotoxicity in human osteoblasts.

A study by Zhao et al., has shown that miR-124 can inhibit viability, promote apoptosis, and impair migration in human endothelial cells [25]. The miR-124 targets are key pro-survival genes, including SphK1 (sphingosine kinase1) [26, 27] CDK6, ROCK1 [20–23], and STAT3 (signal transducers and activators of transcription 3) [28]. The very recent study by Cao et al., has shown that in endothelial cells high glucose (HG) treatment induced circHIPK3 downregulation, causing accumulation of its target miR-124 [14]. Importantly, miR-124 accumulation promoted endothelial cell death and apoptosis [14]. Other studies have also shown that circHIPK3 silencing induced miR-124 accumulation and cancer cell death and apoptosis [20, 23]. Additionally, in the hepatocellular carcinoma cells, miR-124 accumulation following circHIPK3 inhibition induced significant cell apoptosis [13].

In the current study, we show that circHIPK3 possibly acts as the sponge of miR-124 in human osteoblasts. miR-124 levels were significantly increased in circHIPK3-silenced OB-6 cells, but downregulated with circHIPK3 overexpression. Moreover, circHIPK3 downregulation by H_2_O_2_ induced miR-124 accumulation in OB-6 cells and primary human osteoblasts. On the contrary, miR-124 inhibition by a miR-124 inhibitor protected osteoblasts from H_2_O_2_. Forced expression of miR-124, by the miR-124 mimic, induced significant cytotoxicity in human osteoblasts. Importantly, H_2_O_2_ downregulated verified miR-124’s targets, including CDK6 and ROCK1, in human osteoblasts. These results imply that miR-124 accumulation by circHIPK3 downregulation possibly mediated H_2_O_2_-induced cytotoxicity in human osteoblasts.

Together, we show that circHIPK3 downregulation mediates H_2_O_2_-induced cytotoxicity in human osteoblasts. Targeting circHIPK3-miR-124 cascade could be a novel strategy to protect human osteoblasts from oxidative injury.

## MATERIALS AND METHODS

### Reagents

H_2_O_2_ and puromycin were purchased from Sigma-Aldrich Co. (St. Louis, Mo). Fetal bovine serum (FBS), DMEM (Dulbecco's Modified Eagle Medium), antibiotics, and other cell culture reagents were obtained from Gibco-BRL (Grand Island, NY). TRIzol reagent and other RNA assay agents were purchased from Thermo-Fisher (Shanghai, China). Sequences and primers were synthesized by Shanghai Genechem Co. (Shanghai, China). The applied miRNA inhibitors, control miRNA inhibitor, miR-124 mimic and control mimic were purchased from Ambion (Austin, TX). All antibodies were provided by Cell Signaling Tech (Shanghai, China).

### Cell culture

Established OB-6 human osteoblastic cells were provide Dr. Cui [29, 30], cells were cultured as described previously [29, 30]. The primary human osteoblasts were provided by Dr. Ji [17], cultured under a previously-described condition [17, 31]. Primary human osteoblasts at passage 3-10 were utilized for *in vitro* biomedical studies. All protocols were approved by Ethics Committee of authors institutions, and according to the Declaration of Helsinki.

### Human tissues

The lysate samples of necrotic femoral head tissues and the surrounding normal femoral head tissues from ten (10) dexamethasone-taking patients with femoral head resection surgery were provided by Dr. Cui [32]. All clinical investigations were conducted according to the criteria set by the Declaration of Helsinki.

### Quantitative real-time polymerase chain reaction assay (qPCR)

OB-6 cells or the primary human osteoblasts were seeded into six-well plates at 1.5×10^5^ cells per well. Following the treatments, TRIzol reagent was added to extract total cellular RNA. qPCR was performed by a SYBR Green PCR kit (Applied Biosystems, Shanghai, China) under a 7500H FAST Real-Time PCR System (Takara, Osaka, Japan) [33]. Melting curve analysis was always performed to calculate product melting temperature. Using a ^ΔΔ^Ct method, target gene expression was quantified. *U6 RNA* was tested to normalize expression levels of listed genes. All the primers for qPCR assay were purchased from Origene (Beijing, China) Table 1. qPCR primers of CDK6 and ROCK1 were provided by Dr. Wu from Medical School of Nanjing University [20].

**Table 1:** Primers of the qPCR assay in this study.

miR-124-F	5′- GGACTTTCTTCATTCACACCG-3′
miR-124-F	5′- GACCACTGAGGTTAGAGCCA-3′
U6 RNA-F	5′-CTCGCTTCGGCAGCACATATACT-3′
U6 RNA-R	5′-ACGCTTCACGAATTTGCGTGTC-3′
circHIPK3-F	5′-TATGTTGGTGGATCCTGTTCGGCA-3′
circHIPK3-R	5′-TGGTGGGTAGACCAAGACTTGTGA-3′
miR-152-F	5′-TCAGTGCATGACAGAACT-3′
miR-152-F	5′-GAACATGTCTGCGTATCTC-3′
miR-338-F	5′-ATATCCTGGTGCTGAGTG-3′
miR-338-F	5′-GAACATGTCTGCGTATCTC-3′

### Western blotting

At a density of 1.5 × 10^5^ cells per well OB-6 cells or primary human osteoblasts were seeded into six-well plates. Following the treatments, the cell lysis buffer (Biyuntian, Wuxi, China) was added. The lysates (30–40 μg per lane) were separated by 10-12% SDS-PAGE gels, and transferred to polyvinylidene difluoride (PVDF) blots (Millipore, Bedford, MA). After blocking in PBST with 10% non-fat milk, the blots were probed with the designated primary and secondary antibodies. The enhanced chemiluminescence (ECL) reagents (Amersham Bioscience, Piscataway, NJ) were added to visualize the targeted protein signals. Image J software (National Institutes of Health) was utilized for the data quantification.

### Ectopic circHIPK3 overexpression

The lentivirus with pGLV3-U6-GFP-Puro vector encoding circHIPK3 (“LV-circHIPK3”) was provided by Dr. Lu [14], and added to OB-6 cells and primary human osteoblasts. Afterwards, cells were cultured in the fresh complete medium for another 48h. When necessary, puromycin (5 μg/mL) was added to select stable cells for another 10 days. CircHIPK3 overexpression was verified by qPCR.

### circHIPK3 shRNA

Two circHIPK3 shRNAs, with non-overlapping and unique sequence (“S1/S2”), were designed by Shanghai Genechem Co. The shRNA was sub-cloned into a GV248 lentiviral construct to general lentivirus. OB-6 cells and primary human osteoblasts were seeded into the six-well plates at a 50% confluence, and the shRNA lentivirus was added. Afterwards, cells were cultured in the fresh complete medium for another 48h. When necessary puromycin (5 μg/mL) was added to the medium to select stable cells. CircHIPK3 knockdown was confirmed by qPCR.

### Cell viability

At a density of 5 × 10^3^ cells per well OB-6 cells or primary human osteoblasts were seeded into 96-well plates. Following the applied H_2_O_2_ treatment, cell viability was tested by a 3-[4,5-dimethylthylthiazol-2-yl]-2,5 diphenyltetrazolium bromide (MTT) dye assay. At the wavelength of 590 nm MTT optical density(OD) values were tested.

### Cell death assay

At a density of 1.5 × 10^5^ cells per well OB-6 cells or primary human osteoblasts were seeded into six-well plates. Following the treatments, cell death was tested by examining lactate dehydrogenase (LDH) release in the conditional medium, by a simple two-step LDH kit (Takara, Tokyo, Japan). Medium LDH contents were always normalized to the total LDH contents.

### TUNEL [terminal deoxynucleotidyl transferase(TdT)-mediated dUTP nick end labeling] staining

At a density of 5 × 10^4^ cells per well OB-6 cells or primary human osteoblasts were seeded into twelve-well plates. Following the treatments, cells were further stained with TUNEL and DAPI (4',6-diamidino-2-phenylindole, dihydrochloride) dyes. TUNEL ratio (vs. DAPI) was calculated, recording 500 cells of each treatment from five random views (1 : 100 magnification).

### JC-1 assaying of mitochondrial depolarization

In stressed cells mitochondrial depolarization will cause JC-1 aggregating in mitochondria, thereby forming green monomers [34]. OB-6 cells or primary human osteoblasts were seeded into 12-well tissue-culture plates (5 × 10^4^ cells in each well). Following the applied treatments cells were stained with JC-1 (5 μg/mL) and tested immediately by a fluorescence spectrofluorometer at 550 nm. The representative JC-1 images, merging both the green fluorescence image (at 550 nm) and the red fluorescence image (at 650 nm), were presented.

### Annexin V assay

OB-6 cells or primary osteoblasts were seeded into six-well plates (3 × 10^5^ cells per well). Following the applied treatment cells were incubated with Annexin V (10 μg/mL) and PI (10 μg/mL), and analyzed by a fluorescent-activated cell sorting (FACS) machine. The Annexin V ratio was recorded.

### Transfection of miR mimic and miR inhibitors

OB-6 cells and primary human osteoblasts were seeded into the six-well plates at a 40-50% confluence. Cells were transfected with 500 nM of the applied miR inhibitor, control miR inhibitor or miR-124 mimic by Lipofectamine 2000 (Thermo-Fisher) for 24h. The siRNA/mimic transfection was repeated another round (total 48h). Afterwards, miRNA expression was tested by qPCR.

### Statistical analysis

Data were presented as the mean ± standard deviation (SD). ANOVA with multiple comparisons through Bonferroni post-hoc test, analyzed by SPSS version 18.0 (SPSS Co., Chicago, IL), was utilized to test statistical differences. Values of *P* < 0.05 were considered statistically significant.
